# Salivary Microbiota and Network Alterations in Oral Lichen Planus with *Candida* Compared to Healthy Controls

**DOI:** 10.3390/microorganisms14102265

**Published:** 2026-10-05

**Authors:** Hye-Min Ju, Yong-Woo Ahn, Soo-Min Ok, Sung-Hee Jeong, Ji-Young Joo, Hee Sam Na

**Affiliations:** 1 Department of Oral Medicine, Dental and Life Science Institute, School of Dentistry, Pusan National University, Busandaehak-ro 49, Mulgeum-eup, Yangsan-Si 50612, Republic of Korea; jc2wma@pusan.ac.kr (H.-M.J.); ahnyongw@pusan.ac.kr (Y.-W.A.); oksoomin@pusan.ac.kr (S.-M.O.); drcookie@pusan.ac.kr (S.-H.J.); 2 Department of Oral Medicine, Dental Research Institute, School of Dentistry, Pusan National University, Busandaehak-ro 49, Mulgeum-eup, Yangsan-Si 50612, Republic of Korea; 3 Department of Periodontology, Dental Research Institute, Pusan National University Dental Hospital, Yangsan 50612, Republic of Korea; joojy@pusan.ac.kr; 4 Department of Microbiology and Immunology, Chonnam National University Medical School, Hwasun 58128, Republic of Korea; 5 Department of Oral Microbiology, School of Dentistry, Pusan National University, Yangsan 50612, Republic of Korea

**Keywords:** oral lichen planus, *Candida albicans*, salivary microbiome, interkingdom dysbiosis, network analysis, oral microbiota

## Abstract

**Background:** Oral lichen planus (OLP) is a chronic immune-mediated mucosal disorder frequently accompanied by *Candida* colonization. However, the polymicrobial interactions and network alterations underlying this association remain poorly understood. This study aimed to characterize bacterial–fungal community changes and interkingdom network dynamics in OLP patients with *Candida* detection compared to healthy controls. **Methods:** Unstimulated saliva samples were collected from 24 OLP patients and 31 healthy controls. Bacterial 16S rRNA (V1–V2) and fungal ITS2 regions were simultaneously sequenced using Illumina NovaSeq. Taxonomic profiling, diversity analyses, differential analysis, and intermicrobial correlations were analyzed. **Results:** Beta diversity analysis revealed distinct community separation between groups (*p* = 0.001). OLP exhibited increased Bacteroidetes and decreased Firmicutes compared to controls. Health-associated commensals (*Streptococcus salivarius, Rothia mucilaginosa, Haemophilus parainfluenzae, Veillonella* sp. HMT 917) were significantly depleted, while pro-inflammatory anaerobes (*Prevotella, Fusobacterium, Leptotrichia, Selenomonas*) were enriched. At the genus level, *Candida* abundance was modestly higher in the OLP group (1.38%) compared to Healthy (0.94%), and *Candida albicans* was the most prominent fungal species, with a mean relative abundance of 1.22% in OLP compared to 0.91% in Healthy controls, calculated using the same sample-level denominator as the genus-level values above. Network analysis demonstrated that *Candida* occupied a central position with expanded connectivity to disease-associated genera. The disease network showed increased negative correlations and dispersed topology, reflecting inversely correlated taxa and reduced modularity (0.206 vs. 0.260 in healthy), consistent with less distinct community compartmentalization. **Conclusions:** OLP is characterized by compositional dysbiosis and disrupted bacterial–fungal interaction networks. *Candida* may function as a network hub associated with interkingdom dysbiosis in OLP. These findings suggest that microbiome-targeted adjunctive therapies—including antifungals, selective antimicrobials, and probiotics—warrant investigation for managing *Candida*-associated OLP.

## 1. Introduction

Oral lichen planus (OLP) is a chronic inflammatory mucocutaneous disease of unknown etiology that affects approximately 1–2% of the general population, with a higher prevalence in middle-aged women [1]. Clinically, OLP exhibits diverse morphologies—from asymptomatic reticular lesions to painful erosive ulcers—and is considered a potentially malignant disorder due to its association with oral squamous cell carcinoma [2,3]. The pathogenesis of OLP is multifactorial, involving immune dysregulation, genetic predisposition, and environmental triggers [4].

Recent studies have suggested that the oral microbiome may play a critical role in modulating mucosal immune responses and influencing the clinical course of OLP [5,6,7]. Alterations in bacterial community structure, including reduced commensal diversity and increased abundance of pathogenic taxa, have been reported in OLP patients [6]. In addition to bacterial dysbiosis, fungal colonization—particularly by *Candida albicans*—is frequently observed in OLP, especially in erosive forms, and may exacerbate mucosal inflammation [5,8]. While *Candida* overgrowth is often considered a secondary opportunistic event due to impaired epithelial barrier function, emerging evidence suggests it may actively contribute to disease persistence and symptom severity [9].

From an immunopathological perspective, *Candida* colonization in OLP lesions may intensify local inflammatory responses through multiple mechanisms. *C. albicans* can produce virulence factors such as secreted aspartyl proteinases (SAPs) and candidalysin, which damage epithelial cells and activate innate immune receptors including TLR2, TLR4, and dectin-1 [10,11,12]. This activation promotes the release of pro-inflammatory cytokines (e.g., IL-1β, IL-6, TNF-α) and chemokines that recruit neutrophils and T cells to the lesion site [13]. In susceptible hosts, sustained Th1 and Th17 responses contribute to chronic mucosal damage and delayed healing [14]. Furthermore, *Candida*-associated dysbiosis can disrupt bacterial community balance, favoring anaerobic and pro-inflammatory taxa, which may create a self-perpetuating cycle of inflammation and microbial imbalance [15].

Most previous microbiome studies in OLP have focused primarily on either bacterial or fungal communities, with fewer studies integrating both kingdoms simultaneously [16,17,18]. However, the oral microbiome is a complex, multispecies ecosystem in which bacteria and fungi interact through synergistic or antagonistic relationships, potentially influencing disease pathogenesis [18,19]. Network-based analyses can reveal these interkingdom interactions and provide insight into community stability, resilience, and dysbiosis patterns [20]. Despite this, little is known about how co-occurrence patterns of bacterial and fungal taxa are altered in OLP, particularly in cases with *Candida* colonization.

Therefore, this study aimed to comprehensively characterize the bacterial and fungal communities in the saliva of OLP patients with *Candida* detection (OLP) compared to healthy controls (Healthy). Using 16S rRNA and ITS2 amplicon sequencing combined with co-occurrence network analysis, we investigated (i) taxonomic differences in bacterial and fungal composition, (ii) differentially abundant taxa associated with health or disease, and (iii) alterations in microbial interaction networks. These findings may advance our understanding of the role of polymicrobial dysbiosis in OLP pathogenesis and potentially inform future therapeutic strategies targeting the oral microbiome.

## 2. Materials and Methods

### 2.1. Ethics Statement

All subjects provided written consent to participate in the study, and the Institutional Review Board of Pusan National University Dental Hospital (2023-05-021-001) approved the protocol. The study was conducted in accordance with the principles of the Declaration of Helsinki for human studies.

### 2.2. Study Population and Sample Collection

Oral samples were collected from all participants at the Department of Oral Medicine, Pusan National University Dental Hospital, Yangsan, Republic of Korea. Participants were instructed to abstain from eating and performing oral hygiene (such as brushing and flossing) for two hours prior to sampling. They were then asked to rinse their mouths with water and wait at least ten minutes before providing a sample. Saliva was collected by passive drooling into a 2 mL polypropylene tube over a duration of three minutes. Saliva samples from patients with OLP and from healthy volunteers were stored at −80 °C for preservation in the Biobank of Pusan National University Dental Hospital. The samples used in this study were provided by the Biobank of Pusan National University Dental Hospital, a member of the Korea Biobank Network (KBN4_A04). The study included a total of 24 patients with OLP and 31 healthy subjects. OLP was diagnosed according to the modified World Health Organization (WHO) clinical criteria, with histopathological confirmation obtained where clinically indicated. Cases of oral lichenoid lesions were excluded based on clinical assessment by experienced oral medicine specialists, with reference to lesion morphology, distribution pattern, and relevant medical or medication history. Healthy controls were adults with no history of oral mucosal disease, no systemic conditions associated with oral microbiome alteration, and no use of antibiotics, antifungals, immunomodulatory medications, or antiseptic mouthwashes within 4 weeks prior to sampling.

### 2.3. Extraction of Genomic DNA and Next-Generation Sequencing

Saliva samples were suspended in phosphate-buffered saline (PBS), vortexed, and centrifuged. DNA was extracted using a Gram-positive DNA purification kit (Lucigen, Biosearch Technology, Novato, CA, USA) according to the manufacturer’s instructions. Final DNA concentrations were measured using a NanoDrop ND-1000 spectrophotometer (Thermo Fisher Scientific Inc., Waltham, MA, USA), and samples were stored at −80 °C until required. PCR amplification was performed on the V1–V2 and ITS2 regions. The primer sequences used for amplification were as follows: V1-V2-F: 5′-AGA GTT TGA TYM TGG CTC AG-3′; V1-V2-R: 5′-TGC TGC CTC CCG TAG RAG T-3′; ITS2-F: 5′-GCA TCG ATG AAG AAC GCA GC-3′; ITS2-R: 5′-TCC TCC GCT TAT TGA TAT GC-3′ [21]. PCR mixes (20 μL) contained 20 ng of DNA template, equal concentrations of V1–V2 and ITS2 primers (10 pmol), 200 μM dNTPs, and 1 unit of Taq DNA polymerase (Zymo Research, Irvine, CA, USA). Amplification was performed using 24 cycles of 30 s at 94 °C, 30 s at 55 °C, and 30 s at 72 °C, followed by a final 10 min extension at 72 °C. Libraries were prepared using the Herculase II Fusion DNA Polymerase Nextera XT Index Kit V2 (Illumina, San Diego, CA, USA). Purified amplicons were pooled at equimolar concentrations and paired-end sequenced with a NovaSeq 6000 (Illumina, San Diego, CA, USA). Extraction blanks and PCR-negative controls were included in the sequencing run; no significant contamination was detected. No in-run mock community was included; the simultaneous 16S–ITS2 sequencing protocol has been previously validated using the Microbial Community Standard (Zymo Research, Irvine, CA, USA) [21].

### 2.4. Bioinformatic Analysis, Statistical Analysis, and Visualization

Demographic and clinical data were analyzed using SPSS software (version 23.0; SPSS Inc., Chicago, IL, USA). Statistical significance was set at *p* < 0.05. The Shapiro–Wilk test was performed to assess data normality. Fisher’s exact test was used to compare sex distribution between groups. Differences in age and number of teeth were analyzed using independent-samples t-tests after confirming normal distribution. Variables with very low frequencies (e.g., smoking, alcohol drinking, diabetes) were described descriptively without inferential statistics.

Basic microbiome analysis was performed using QIIME2 (version 2024.10) [22] and associated plugins. Primer sequences were removed by the sequencing provider (Macrogen, Seoul, Republic of Korea) prior to receipt of the raw data. DADA2 (Divisive Amplicon Denoising Algorithm 2) was then applied to denoise, join the paired reads, and remove chimeras. Low-quality 5′ bases were trimmed 8 bp from both forward and reverse reads (--p-trim-left-f 8, --p-trim-left-r 8). Reads were truncated to 248 bp (forward) and 245 bp (reverse; --p-trunc-len-f 248, --p-trunc-len-r 245), denoised into ASVs, and chimeric sequences removed using the consensus method (Supplementary Table S1). Chao1 and Shannon indices were used to measure alpha diversity, and the Mann–Whitney U test was used to determine the significance of differences in alpha diversity. To assign taxonomy to unique representative sequences, a pre-trained Naïve Bayes classifier was used with the eHOMD (v15.1) [23] and UNITE (v9.0) [24] databases. To adjust the relative contribution of fungal reads to the combined feature table, read counts of taxa assigned to fungi were reduced by a factor of 20 [21]. The resulting fractional counts were rounded to the nearest integer prior to all downstream analyses, including alpha diversity estimation. Relative abundances were then calculated from the combined corrected feature table, enabling cross-kingdom comparison as previously validated [21]. To test the differential abundance of bacterial species, linear discriminant analysis effect size (LEfSe) (v1.10.1) [25] with an LDA score threshold of 2.0, and ALDEx2 (v1.32.0) [26] using 128 Monte Carlo Dirichlet instances with the inter-quartile log-ratio (iqlr) denominator and Benjamini–Hochberg correction (wi.eBH < 0.05), were applied; taxa significant in both methods were considered the final significant taxa (Supplementary Table S2). To visualize internal interactions and further characterize the microbial community, Sparse Correlations for Compositional data (SparCC) (v1.1.3) [27] was used to calculate the pairwise correlation coefficients, with significance estimated using 1000 bootstrap iterations. Taxa present in fewer than 20% of samples were excluded prior to network construction. Pairwise correlations were estimated using the SparCC algorithm implemented via FastSpar, correcting for compositional bias. Zero counts were adjusted by adding a pseudocount prior to log-ratio transformation. Basis variances were calculated over 20 outer iterations with a correlation exclusion threshold of 0.1. Statistical significance of edges was estimated using 1000 bootstrap iterations; two-sided empirical *p*-values were adjusted using Benjamini–Hochberg FDR correction. Edges were retained when both |r| ≥ 0.30 and FDR q < 0.05 were satisfied. The network was then visualized with Cytoscape (v3.10.4) [28], and topological metrics were computed (Supplementary Table S3).

### 2.5. Data Availability

The raw sequencing data have been deposited at NCBI GenBank under BioProject ID PRJEB124089. Note: Section 2.4 contains all bioinformatic and statistical analyses, including taxonomic profiling, diversity analyses, differential abundance testing (LEfSe and ALDEx2), and co-occurrence network construction (SparCC/Cytoscape).

## 3. Results

### 3.1. Patient Characteristics

Table 1 presents the demographic and clinical characteristics of the study participants. The mean age was notably higher in the OLP group (71.67 ± 9.30 years) compared to the healthy controls (63.68 ± 13.59 years), with a mean difference of approximately 8.0 years (*p* = 0.017). The sex distribution did not differ significantly between the groups (Healthy: 8 males/23 females; OLP: 6 males/18 females, *p* = 1.000). The average number of teeth was comparable between the groups (24.32 ± 6.67 in Healthy vs. 22.67 ± 6.75 in OLP, *p* = 0.368). None of the participants reported cigarette smoking. One participant in the OLP group reported alcohol consumption, whereas none did in the Healthy group. Diabetes was reported in one Healthy subject and in two subjects in the OLP group.

### 3.2. Diversity and Abundance of Bacterial and Fungal Communities in Healthy and OLP Groups

**Figure 1:**
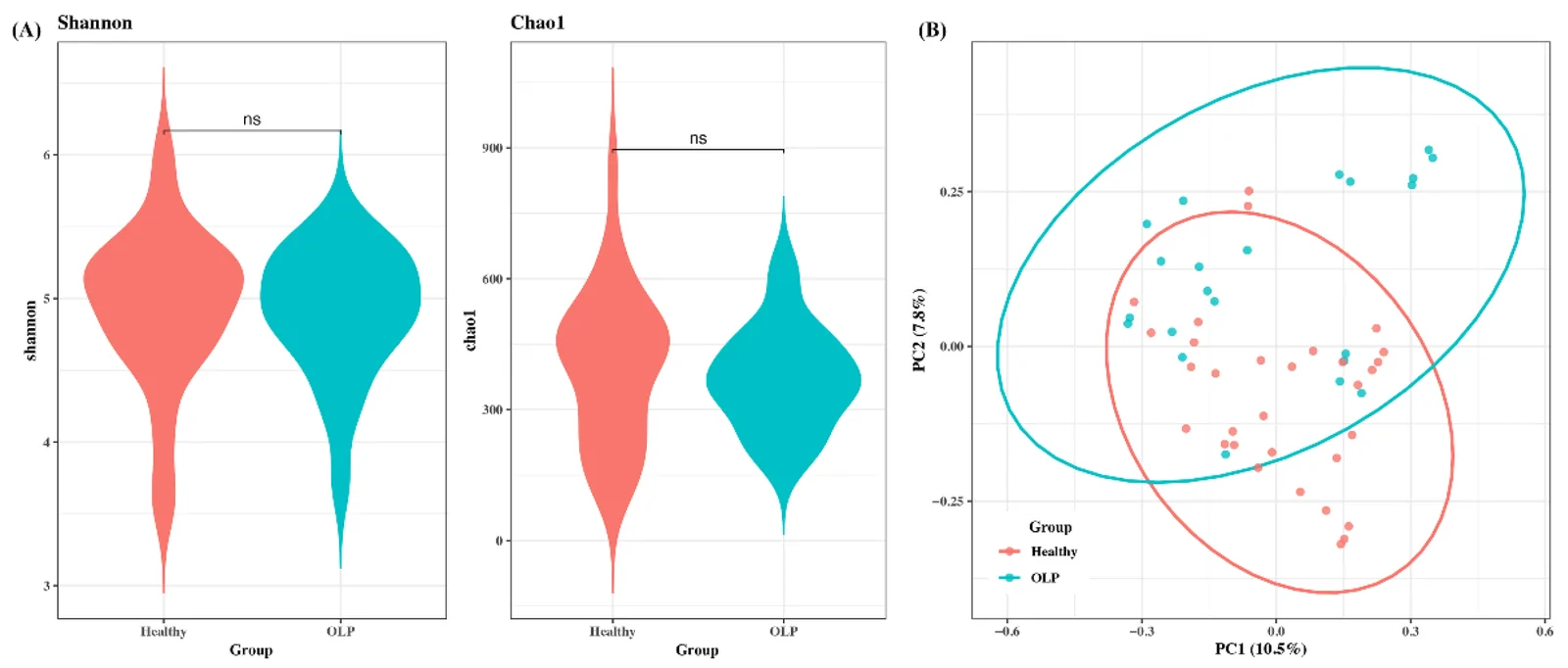
Combined bacterial and fungal microbiota comparisons between healthy and OLP groups (after 20-fold fungal read correction). (**A**) Alpha diversities of saliva samples (Shannon index and Chao1 index); ns, not significant. (**B**) Beta diversity of salivary samples based on Bray–Curtis distance. PERMANOVA (pseudo-F = 2.233, R^2^ = 0.0390, *p* = 0.001, 999 permutations) was used to identify intergroup differences.

The alpha diversity of the combined bacterial–fungal microbiota (calculated from the combined corrected feature table after 20-fold fungal read correction) was evaluated using the Shannon and Chao1 indices. No significant differences were observed between the Healthy and OLP groups in either index (Figure 1A). This finding indicates that overall within-sample species richness and evenness were comparable between groups; however, the absence of a significant difference in alpha diversity does not imply community equivalence, as these indices do not capture compositional identity. Beta diversity analysis based on Bray–Curtis distance and visualized through principal coordinates analysis (PCoA) revealed a significant difference in overall microbial community structure between the two groups (PERMANOVA: pseudo-F = 2.233, R^2^ = 0.0390, *p* = 0.001, 999 permutations) (Figure 1B), indicating that the two communities differ in their overall composition.

**Figure 2:**
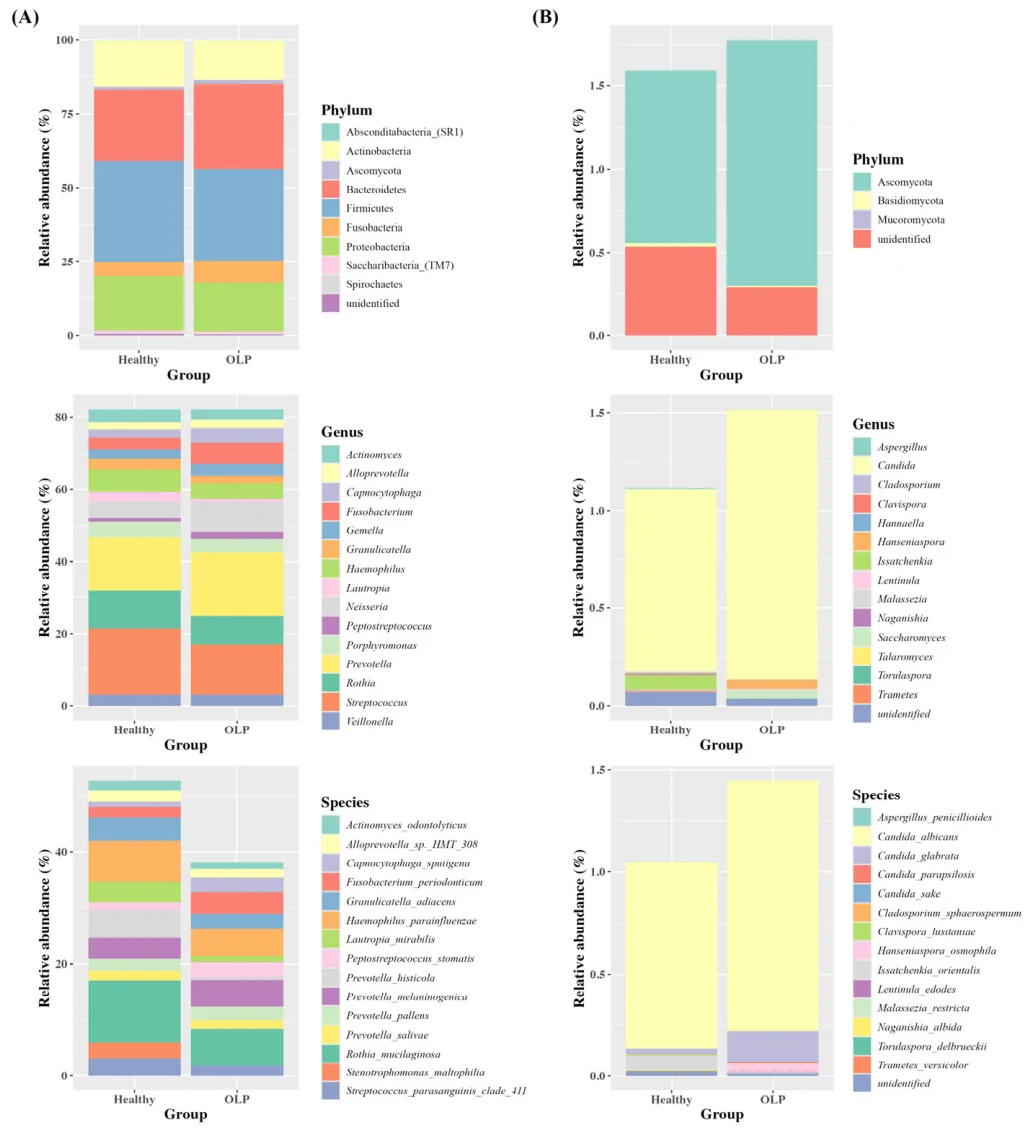
Relative abundances of microbial taxa in salivary samples of healthy and OLP groups. (**A**) Bacterial relative abundance at the phylum, genus, and species levels. (**B**) Fungal relative abundance at the phylum, genus, and species levels. The relative abundance of the top 15 most abundant taxa is plotted for each group.

The bacterial community at the phylum level was dominated by Firmicutes, Bacteroidetes, Proteobacteria, and Actinobacteria in both groups, together accounting for over 95% of all sequences (Figure 2A). Bacteroidetes were relatively more abundant in the OLP group (28.5%) compared to the Healthy group (23.9%), whereas Firmicutes showed a modest decrease (OLP: 31.1%; Healthy: 34.2%).

At the genus level, *Prevotella* and *Streptococcus* were among the most abundant genera. *Prevotella* was more enriched in the OLP group (13.9%) than in Healthy (11.0%), while *Streptococcus* showed a marked decrease (10.5% vs. 17.6%). Similarly, *Fusobacterium* was more abundant in the OLP group (7.3%) than in Healthy (4.8%), whereas *Rothia*, a common commensal genus, was less abundant (5.2% vs. 9.5%).

In terms of fungal composition, Ascomycota was the predominant phylum in both groups and was slightly more abundant in OLP (1.48%) than in Healthy controls (1.04%). At the genus level, *Candida* was dominant and more abundant in the OLP group (1.38%) compared to Healthy (0.94%); these values represent mean relative abundance calculated using a single sample-level denominator prior to taxonomic aggregation. At the species level, *Candida albicans* was the most prominent species in the OLP group (Figure 2B).

### 3.3. Differentially Abundant Taxa and Co-Occurrence Network Analysis Between Healthy and OLP Groups

**Figure 3:**
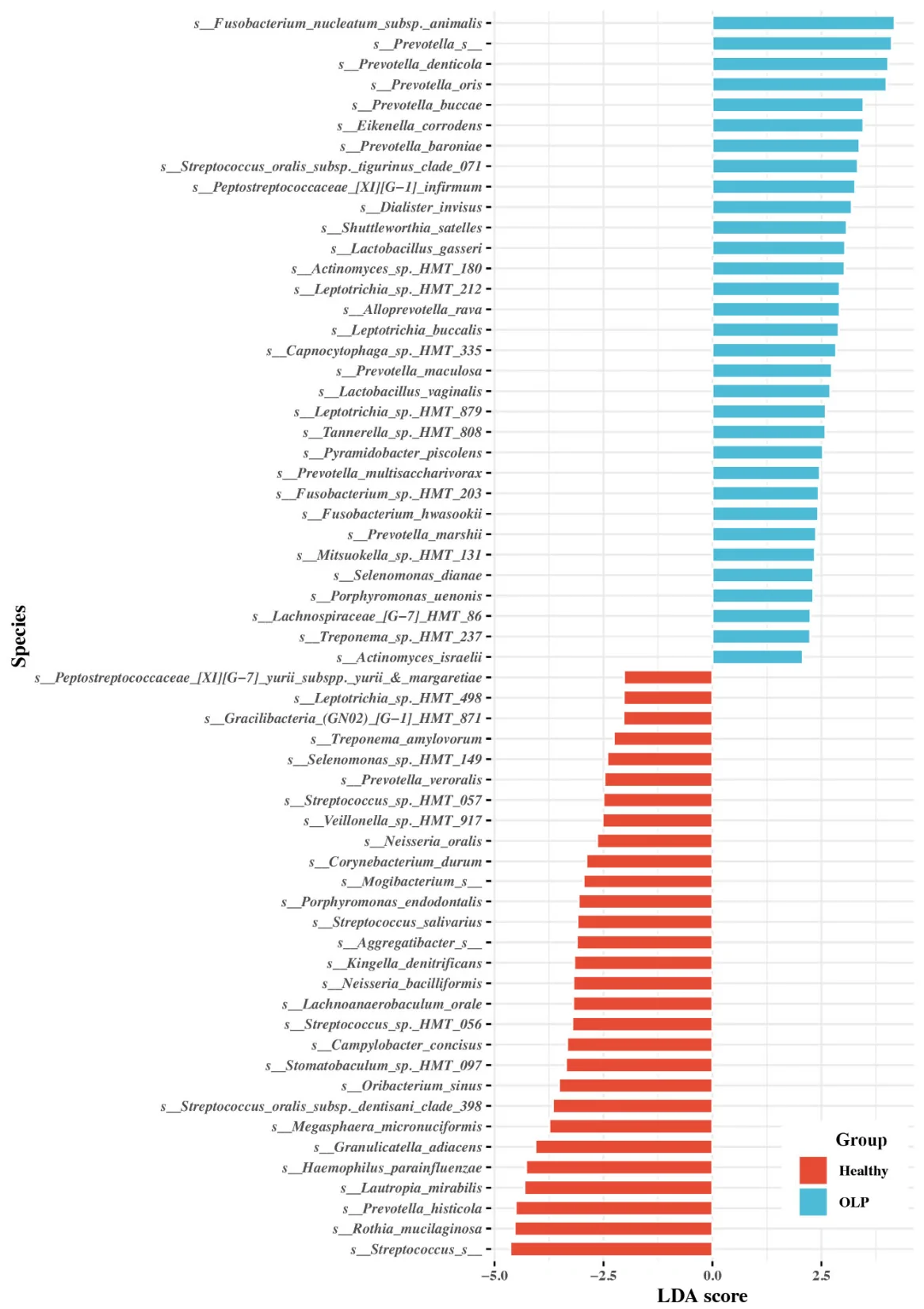
Comparisons of microbiota abundance in healthy and OLP samples showing significant differences. This analysis was performed using linear discriminant analysis (LDA) and effect size analysis (LEfSe). Healthy- and OLP-associated bacterial species with an LDA score > 2.0 at the species level were plotted.

**Figure 4:**
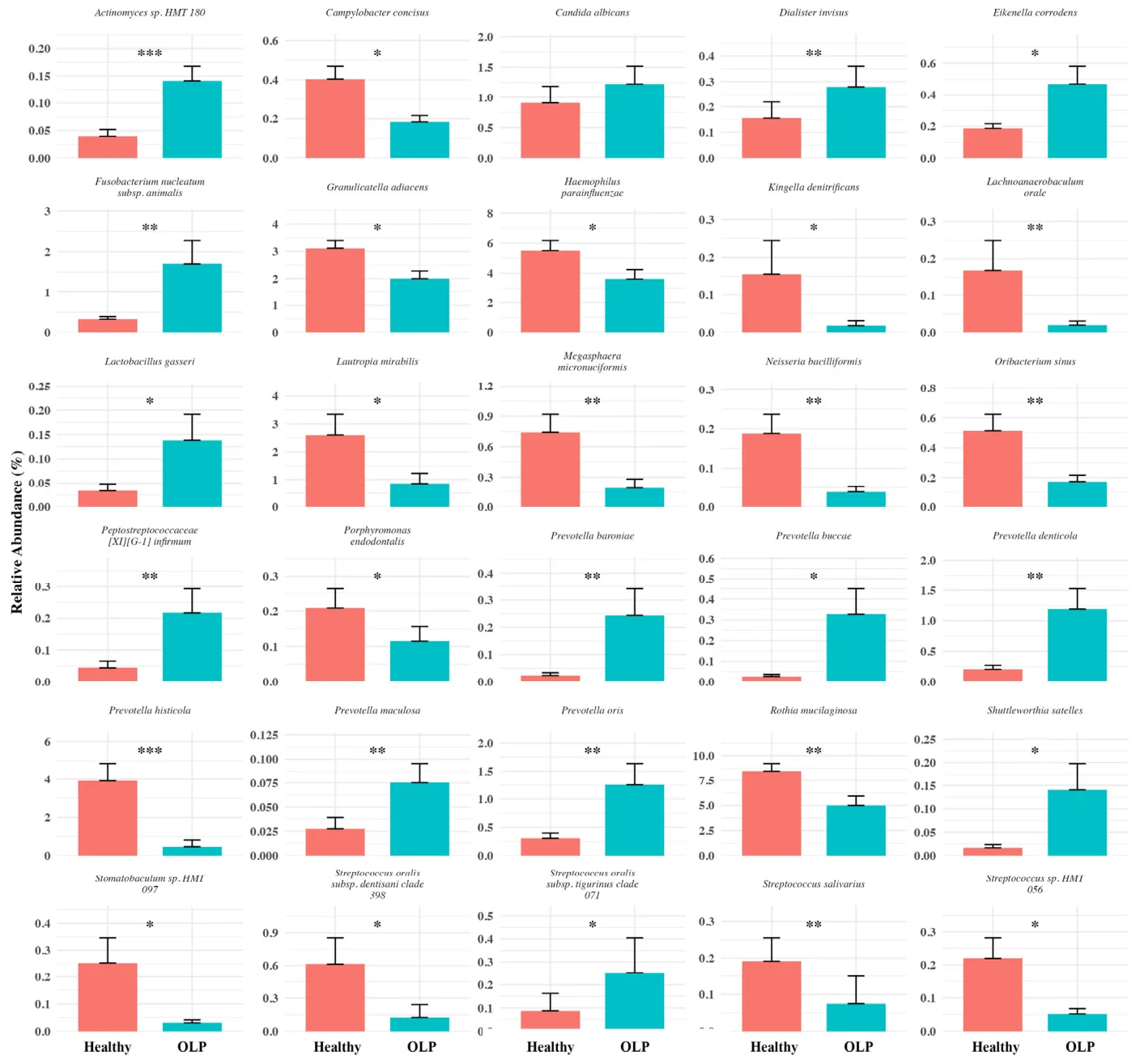
Relative abundance of selected significant bacterial species. The significance of intergroup differences was determined using Tukey’s post hoc test. * *p* < 0.05; ** *p* < 0.01; *** *p* < 0.001.

LEfSe and ALDEx2 were used to evaluate differences in bacterial species abundance between the Healthy and OLP groups. Several species were identified as significantly different between the two groups (Figure 3; full LEfSe and ALDEx2 outputs and their intersection are provided in Supplementary Table S2). The Healthy group had higher relative abundances of *Streptococcus salivarius*, *Haemophilus parainfluenzae*, *Rothia mucilaginosa*, and *Veillonella* sp. HMT 917. In contrast, the OLP group exhibited increased relative abundances of several pro-inflammatory or dysbiosis-associated taxa, including *Prevotella baroniae*, *Prevotella maculosa*, *Leptotrichia buccalis*, *Fusobacterium nucleatum* subsp. *animalis*, and *Selenomonas dianae*, along with enriched genera such as *Prevotella*, *Leptotrichia*, *Fusobacterium*, and *Selenomonas* (Figure 3). For illustrative purposes, Tukey’s post hoc test was applied to selected taxa from the LEfSe–ALDEx2 intersection (Figure 4); this is not intended as independent statistical confirmation, as multiplicity across the full taxon set is not addressed. The OLP group exhibited significantly higher abundances of *Dialister invisus* and *Prevotella denticola*, while *Haemophilus parainfluenzae* and *Streptococcus salivarius* were significantly more abundant in the Healthy group.

**Figure 5:**
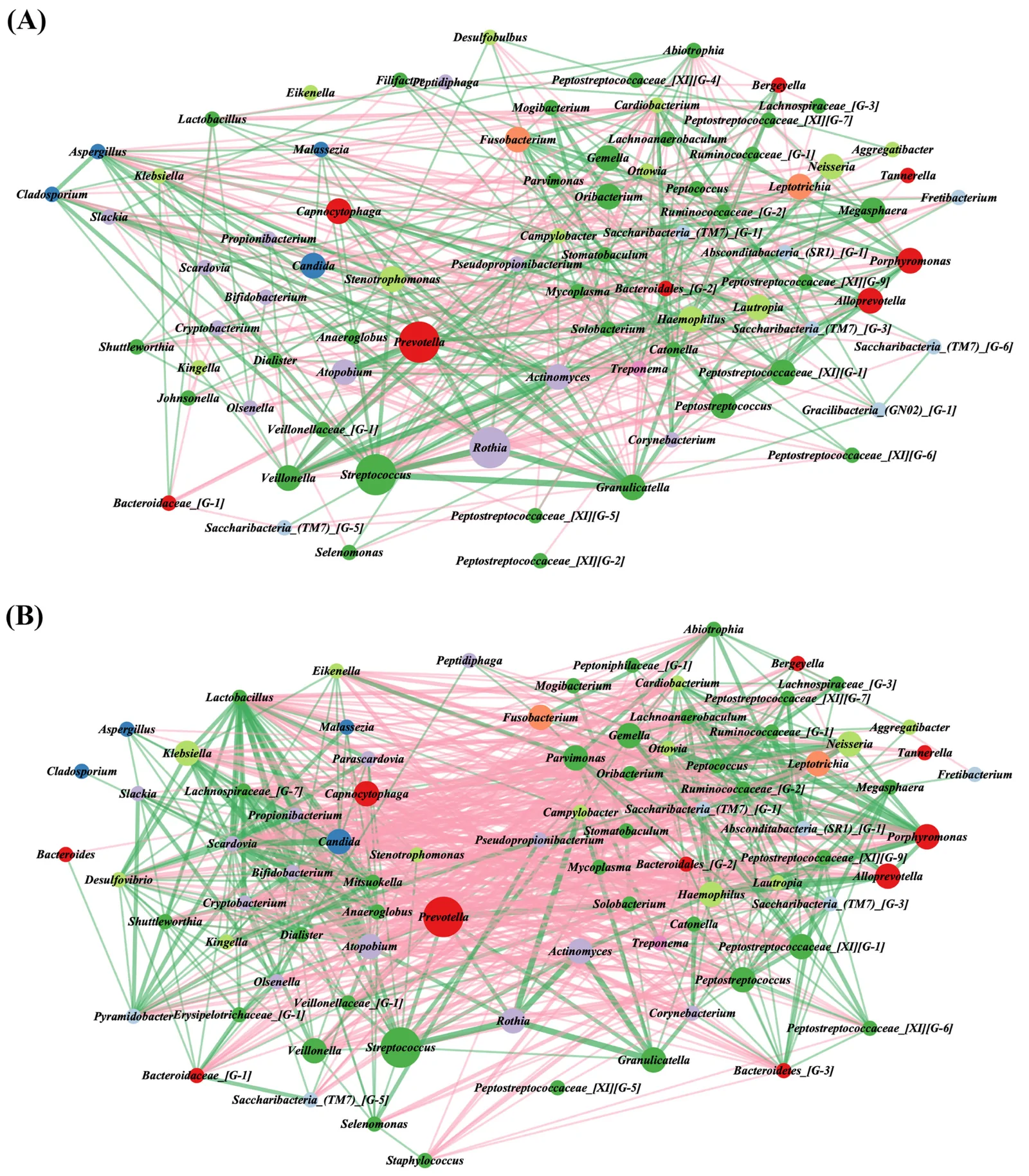

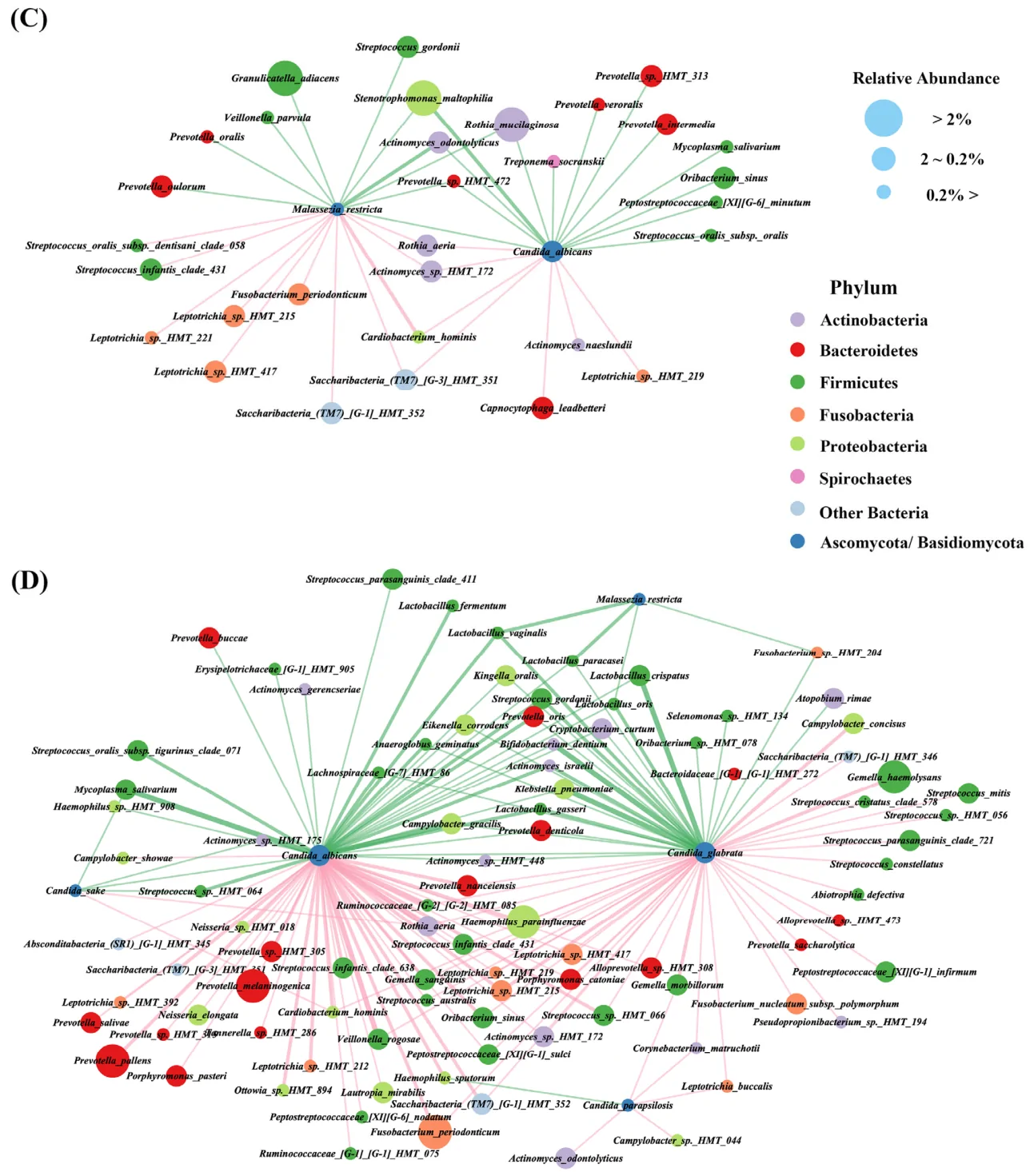
Salivary microbiome network analysis. (**A**) Healthy group and (**B**) OLP group at the genus level. (**C**) First-degree network with fungi in the healthy group and (**D**) in the OLP group at the species level. Network nodes represent genera with size proportional to relative abundance, and node colors indicate phylum-level taxonomy according to the legend. Edges (connections) between nodes indicate significant correlations between genera. Positive correlations are indicated by green lines and negative correlations by red lines.

To further assess microbial interactions, genus-level co-occurrence network analysis was conducted (Figure 5). Network nodes represent genera with size proportional to relative abundance, and node colors indicate phylum-level taxonomy. Edges represent significant correlations (green for positive, red for negative). In the Healthy group (Figure 5A), health-associated genera—including *Streptococcus*, *Rothia*, and *Veillonella*—formed a densely interconnected core with predominantly positive correlations (green edges), consistent with a stable, mutualistic co-occurrence pattern. The network structure was cohesive, with limited negative interactions. In contrast, the OLP group (Figure 5B) exhibited a fundamentally altered network topology. Disease-associated genera such as *Prevotella* and *Candida* showed increased node size and network centrality. The network displayed substantially more negative correlations and a more dispersed topology compared to the healthy network, with reduced modularity (0.206 vs. 0.260), indicating less distinct community compartmentalization. Although OLP had fewer connected components (4 vs. 9 in healthy), a higher proportion of nodes were incorporated into a single large component (83/86 vs. 80/88), reflecting a network reorganization rather than simple fragmentation.

To specifically examine *Candida*-centered interactions, first-degree neighbor networks were constructed (Figure 5C,D). In the Healthy group (Figure 5C), *Candida* occupied a peripheral network position (degree = 5, betweenness centrality = 0.002 in the genus-level network; Supplementary Table S3), with connections predominantly to commensal genera. At the species level, *Candida albicans* showed a degree of 20 in the *Candida*-centered sub-network. In stark contrast, the OLP group (Figure 5D) revealed that *Candida* dramatically expanded its network (*Candida albicans* degree = 70 at species level vs. 20 in healthy; genus-level *Candida* degree = 29 vs. 5; Supplementary Table S3), establishing extensive connections with pathogenic taxa including *Prevotella*, *Fusobacterium*, *Leptotrichia*, and *Streptococcus*.

## 4. Discussion

The present study provides novel insights into the polymicrobial nature of OLP by simultaneously characterizing bacterial and fungal communities and their interaction networks in patients with *Candida* detection. Our most significant finding is that OLP is characterized not only by taxonomic shifts in individual microbial members but also by fundamental alterations in microbial network architecture, suggesting that interkingdom dysbiosis may play an active role in disease pathogenesis rather than being a mere consequence of mucosal inflammation.

At the phylum level, we observed an increased abundance of Bacteroidetes (28.5% vs. 23.9%) and decreased Firmicutes (31.1% vs. 34.2%) compared to healthy controls. This shift is consistent with dysbiotic patterns reported in other chronic inflammatory oral conditions, including periodontitis and oral squamous cell carcinoma [16,17].

At the genus and species levels, differential analysis identified several key discriminatory taxa. Health-associated commensals—including *Streptococcus salivarius*, *Haemophilus parainfluenzae*, *Rothia mucilaginosa*, and *Veillonella* sp. HMT 917—were significantly depleted in OLP patients. These early colonizers maintain oral microbial homeostasis through competitive exclusion, bacteriocin production, and pH modulation [18,19]. The reduction in *Rothia mucilaginosa* is consistent with previous OLP studies [5,6] and is particularly significant given its role in nitrate reduction and anti-inflammatory nitric oxide homeostasis [20]. Depletion of *Veillonella* species, which metabolize streptococcal lactate and maintain pH balance, indicates disruption of key commensal partnerships [29].

Conversely, the OLP group exhibited significant enrichment of proteolytic and pro-inflammatory anaerobes, particularly *Prevotella baroniae*, *Prevotella maculosa*, *Fusobacterium nucleatum* subsp. *animalis*, *Leptotrichia buccalis*, and *Selenomonas dianae*. These organisms, frequently associated with periodontal disease [6,17], produce lipopolysaccharides that activate TLR4 signaling and induce pro-inflammatory cytokines [30]. Proteolytic enzymes from *Prevotella* can degrade host immunoglobulins, facilitating immune evasion [31], while *Fusobacterium* species produce short-chain fatty acids that impair epithelial barrier function [18,32]. The presence of *Dialister invisus* further supports the dysbiotic nature of the OLP microbiome [6]. In contrast, *Campylobacter concisus* showed higher relative abundance in healthy controls (Supplementary Table S2) and is therefore not interpreted as a dysbiotic marker in this cohort. Of note, the direction of association for individual taxa should be interpreted in conjunction with the figures, as some organisms detected in OLP samples may not show unidirectional enrichment.

A striking finding of our study was that, at the species level, *Candida albicans* was the most prominent fungal species in the OLP group. While genus-level *Candida* abundance showed modest quantitative differences between groups (1.38% vs. 0.94%), its central network positioning and expanded connectivity with disease-associated bacteria suggest a functional rather than purely quantitative role in disease pathogenesis. While fungal colonization in OLP has been documented [8,9], our integrated analysis provides novel insights into interkingdom interactions. *C. albicans* possesses virulence factors that directly contribute to epithelial damage and inflammation. Secreted aspartyl proteinases (SAPs) degrade E-cadherin and occludin, compromising barrier integrity [10], while candidalysin triggers NLRP3 inflammasome activation and IL-1β release [11]—particularly relevant given elevated IL-1β levels in OLP [13]. Fungal cell wall components engage pattern recognition receptors (dectin-1, TLR2, TLR4), initiating neutrophil recruitment and inflammatory responses [12].

The simultaneous enrichment of *Candida* and specific bacterial taxa suggests potential synergistic interkingdom interactions that extend beyond simple co-occurrence. Our network analysis provides quantitative evidence for this synergy: while genus-level *Candida* abundance differences were modest, the dramatic expansion of *Candida*’s network connectivity in OLP patients—from peripheral commensal to central hub—demonstrates its functional rather than purely quantitative role in orchestrating polymicrobial dysbiosis. This bidirectional synergy may explain why *Candida*-colonized OLP lesions are more symptomatic and treatment-resistant [8,9].

Co-occurrence network analysis revealed fundamental alterations in microbial community structure and interaction patterns. The healthy network (Figure 5A) comprised 88 nodes and 404 edges (density = 0.106, mean degree = 9.18, clustering coefficient = 0.386, modularity = 0.260; Supplementary Table S3), with core commensal genera (*Streptococcus* (degree = 29), *Rothia* (degree = 18), *Veillonella* (degree = 21)) forming a tightly integrated hub with predominantly positive correlations (green edges). This topology reflects a stable, mutualistic ecosystem with high functional redundancy and resilience to perturbation [20,33]. The positive correlations among health-associated commensals are consistent with their known roles in facilitating nutrient cycling, pH regulation, and colonization resistance against pathogens [29,34].

In stark contrast, the OLP network (Figure 5B) displayed dysbiotic hallmarks: a substantially reorganized structure comprising 86 nodes and 714 edges (network density = 0.195, mean degree = 16.60, global clustering coefficient = 0.441, modularity = 0.206; Supplementary Table S3). Disease-associated taxa, including *Candida* and *Prevotella* species, occupied visually central positions (Figure 5B); for *Candida*, node-level metrics showed increased connectivity (degree = 29 vs. 5 in healthy; betweenness centrality = 0.012 vs. 0.002; Supplementary Table S3). The network showed a marked increase in negative correlations (red edges) and reduced modularity (0.206 vs. 0.260)—patterns consistent with, though not direct measurements of, altered interaction dynamics [20,35]. Large *Prevotella* nodes reflect their elevated abundance in disease, while *Candida*’s prominent positioning despite modest quantitative abundance underscores its functional significance as a network hub. This dispersed topology with antagonistic interactions destabilizes community structure, reduces functional redundancy, and increases susceptibility to further dysbiosis [20,36,37].

The first-degree neighbor analysis (Figure 5C,D) provides mechanistic insight into the role of fungi in disease pathogenesis. In health (Figure 5C), *Candida* maintains limited, predominantly positive connections to commensal genera, consistent with its status as a controlled commensal. However, in OLP (Figure 5D), *Candida* dramatically expanded its network, establishing extensive connections across multiple phyla—including *Prevotella* species, *Fusobacterium* and *Leptotrichia*, and various *Streptococcus* species. This transformation from peripheral commensal to central network hub suggests *Candida* may function as a network hub potentially associated with interkingdom dysbiosis, consistent with its known virulence mechanisms reported in the literature [15,18]. Whether this represents a causal keystone role or a correlational network signature requires longitudinal or interventional evidence.

The central positioning of *Candida* and its expanded connectivity suggest it may function as an organizing principle for the dysbiotic bacterial community. *C. albicans* creates microenvironments favorable for anaerobes by reducing oxygen tension and producing metabolic byproducts (lactate, ethanol, acetate) that serve as carbon sources for *Prevotella* and *Fusobacterium* [15,18]. In vitro studies demonstrate that *Candida* biofilms enhance periodontal pathogen virulence through metabolic cross-feeding and co-aggregation [38]. Reciprocally, bacteria promote fungal hyphal formation through quorum-sensing molecules and protect fungal cells via biofilm encapsulation [18,39]. *Streptococcus gordonii* physically interacts with *C. albicans* through Als3–SspB binding, enhancing adhesion and mixed-species biofilm formation [40]. The loss of positive correlations among health-associated commensals indicates disruption of beneficial partnerships that normally maintain oral homeostasis [34]. These network alterations parallel findings in other polymicrobial oral diseases [6,20], supporting the concept that network topology—not merely taxonomic composition—determines disease risk, severity, and treatment responsiveness.

### 4.1. Clinical and Therapeutic Implications

Our findings have several translational implications. First, the association between *Candida* and bacterial dysbiosis suggests that comprehensive antimicrobial approaches may be warranted. While topical corticosteroids remain the cornerstone of OLP therapy [4], adjunctive antifungal treatment has shown promise in *Candida*-positive erosive OLP [8,9,41]. Prior clinical studies suggest that combined corticosteroid–antifungal therapy may offer benefit in *Candida*-positive OLP [41], and our findings raise the hypothesis that targeting dysbiotic bacteria (e.g., with chlorhexidine) could be explored in future intervention studies.

Second, depletion of health-associated commensals suggests microbiome restoration potential. Probiotic supplementation with *Streptococcus salivarius* or *Lactobacillus reuteri* could potentially support commensal restoration—a hypothesis that warrants testing in future clinical trials [18,42], while prebiotics such as xylitol may selectively promote commensal growth [43]. Third, network-based profiling may offer exploratory value for understanding disease heterogeneity; however, individual-level prognostic applications require validation in prospective studies with larger cohorts before clinical stratification can be considered [44].

### 4.2. Limitations and Future Directions

Several limitations should be acknowledged. The cross-sectional design precludes causal inference; longitudinal studies tracking microbiome dynamics from health to disease are needed [45]. The substantial age difference between groups (OLP: 71.67 years vs. Healthy: 63.68 years; mean difference ~8.0 years) represents a notable limitation that cannot be dismissed as negligible. Although this imbalance reflects OLP’s natural epidemiology—predominantly affecting older adults with peak incidence in the sixth–seventh decades [1]—and recruiting closely age-matched controls proved challenging due to comorbidities requiring exclusion, we acknowledge that age-adjusted analyses and sensitivity analyses within the overlapping age range were not performed in the current study. While aging influences oral microbiome composition [46], our findings are directionally consistent with age-matched OLP studies [5,6], suggesting that disease-related signatures may contribute substantially to the observed differences. Nevertheless, stricter age-matching in future studies would strengthen the conclusions.

Salivary sampling may not fully capture lesion-site microbiota [47]. Future studies should include matched saliva and lesional samples, as well as an OLP group without *Candida* to distinguish *Candida*-specific from general OLP-associated microbial shifts, as the absence of a *Candida*-negative OLP comparator group precludes isolation of *Candida*-specific contributions to the observed dysbiosis [48]. The modest sample size (*n* = 24 OLP, *n* = 31 Healthy) limited subgroup analyses; larger multicenter studies would enable validation across populations [49].

## 5. Conclusions

This integrated bacterial–fungal microbiome analysis reveals distinct compositional alterations and disrupted interaction networks in OLP patients with *Candida* colonization. The enrichment of pro-inflammatory anaerobes alongside *Candida albicans*, coupled with depletion of health-associated commensals and network reorganization, supports an interkingdom dysbiosis model in OLP pathogenesis. *Candida* colonization is associated with expanded network connectivity and may play a role beyond passive opportunism—a hypothesis that requires longitudinal and mechanistic validation.

Our results suggest that microbiome-targeted interventions—antifungal therapy, selective antimicrobials, and probiotics—represent promising avenues warranting investigation as adjuncts to conventional immunomodulatory treatment in future appropriately designed trials. Network-based profiling may contribute to understanding disease heterogeneity, though individual-level stratification applications require prospective validation. Future longitudinal and mechanistic studies incorporating multi-omics approaches are essential to fully elucidate the causal role of polymicrobial dysbiosis and to develop effective microbiome-based therapeutic strategies for this challenging chronic mucosal disease.

## Figures and Tables

**Table 1:** Patient characteristics.

Characteristics	HC (*n* = 31)	OLP (*n* = 24)	*p*-Value
Age (mean ± SD)	63.68 ± 13.59	71.67 ± 9.30	0.017
Male/female	8/23	6/18	1.000
No. of teeth (mean ± SD)	24.32 ± 6.67	22.67 ± 6.75	0.368
Cigarette smoking	0	0	
Alcohol drinking	0	1	
Diabetes	1	2	

## Data Availability

The raw sequencing data have been deposited at NCBI GenBank under BioProject ID PRJEB124089.

## Supplementary Materials

The following supporting information can be downloaded at: https://www.mdpi.com/article/10.3390/microorganisms14102265/s1, Table S1: Per-sample sequencing read retention statistics before and after DADA2 denoising, including demographic and *Candida* detection status for each participant; Table S2: Significant bacterial taxa identified by LEfSe and ALDEx2 differential abundance analysis, including the intersection list used to generate Figure 3 and Figure 4; Table S3: Quantitative co-occurrence network metrics (global and node-level) for the healthy and OLP genus-level networks and their respective *Candida*-centered first-degree neighbor sub-networks.

## Abbreviations

The following abbreviations are used in this manuscript:

OLP	Oral lichen planus
HC	Healthy controls
rRNA	Ribosomal ribonucleic acid
ITS2	Internal transcribed spacer 2
DNA	Deoxyribonucleic acid
PCR	Polymerase chain reaction
PBS	Phosphate-buffered saline
dNTPs	Deoxynucleotide triphosphates
NGS	Next-generation sequencing
QIIME2	Quantitative Insights Into Microbial Ecology 2
DADA2	Divisive Amplicon Denoising Algorithm 2
eHOMD	expanded Human Oral Microbiome Database
UNITE	User-friendly Nordic ITS Ectomycorrhiza Database
LEfSe	Linear discriminant analysis effect size
LDA	Linear discriminant analysis
ALDEx2	ANOVA-Like Differential Expression 2
SparCC	Sparse Correlations for Compositional data
PCoA	Principal coordinates analysis
SPSS	Statistical Package for the Social Sciences
SD	Standard deviation
SAPs	Secreted aspartyl proteinases
TLR	Toll-like receptor
IL	Interleukin
TNF-α	Tumor necrosis factor alpha
Th	T helper cell
NLRP3	NLR family pyrin domain containing 3
NCBI	National Center for Biotechnology Information
